# Genome-wide association of sleep and circadian phenotypes

**DOI:** 10.1186/1471-2350-8-S1-S9

**Published:** 2007-09-19

**Authors:** Daniel J Gottlieb, George T O'Connor, Jemma B Wilk

**Affiliations:** 1 The National Heart, Lung, and Blood Institute's Framingham Heart Study, Framingham, MA, USA; 2 Boston University School of Medicine, 715 Albany Street, Boston, MA 02118, USA; 3 VA Boston Healthcare System, 1400 VFW Parkway, West Roxbury, MA 02130, USA

## Abstract

**Background:**

Numerous studies suggest genetic influences on sleepiness and circadian rhythms. The Sleep Heart Health Study collected questionnaire data on sleep habits and sleepiness from 2848 Framingham Heart Study Offspring Cohort participants. More than 700 participants were genotyped using the Affymetrix 100K SNP GeneChip, providing a unique opportunity to assess genetic linkage and association of these traits.

**Methods:**

Sleepiness (defined as the Epworth Sleepiness Scale score), usual bedtime and usual sleep duration were assessed by self-completion questionnaire. Standardized residual measures adjusted for age, sex and BMI were analyzed. Multipoint variance components linkage analysis was performed. Association of SNPs to sleep phenotypes was analyzed with both population-based and family-based association tests, with analysis limited to 70,987 autosomal SNPs with minor allele frequency ≥10%, call rate ≥80%, and no significant deviation from Hardy-Weinberg equilibrium (p ≥ 0.001).

**Results:**

Heritability of sleepiness was 0.29, bedtime 0.22, and sleep duration 0.17. Both genotype and sleep phenotype data were available for 749 subjects. Linkage analysis revealed five linkage peaks of LOD >2: four to usual bedtime, one to sleep duration. These peaks include several candidate sleep-related genes, including *CSNK2A2*, encoding a known component of the circadian molecular clock, and *PROK2*, encoding a putative transmitter of the behavioral circadian rhythm from the suprachiasmatic nucleus. Association tests identified an association of usual bedtime with a non-synonymous coding SNP in *NPSR1 *that has been shown to encode a gain of function mutation of the neuropeptide S receptor, whose endogenous ligand is a potent promoter of wakefulness. Each copy of the minor allele of this SNP was associated with a 15 minute later mean bedtime. The lowest p value was for association of sleepiness with a SNP located in an intron of *PDE4D*, which encodes a cAMP-specific phosphodiesterase widely expressed in human brain. Full association results are posted at http://www.ncbi.nlm.nih.gov/projects/gap/cgi-bin/study.cgi?id=phs000007.

**Conclusion:**

This analysis confirms prior reports of significant heritability of sleepiness, usual bedtime, and usual sleep duration. Several genetic loci with suggestive linkage to these traits are identified, including linkage peaks containing circadian clock-related genes. Association tests identify *NPSR1 *and *PDE4D *as possible mediators of bedtime and sleepiness.

## Background

Daytime sleepiness is a common symptom, experienced at least 3 days per week by 29% of respondents in a recent poll of U.S. adults [1]. Sleepiness is a major cause of motor vehicle and occupational accidents, impaired social function, and reduced quality of life. Within individuals, the level of sleepiness is modulated by a combination of homeostatic (duration of wakefulness) and circadian (time of day) factors [2]. While behavioral factors and sleep disorders contribute to daytime sleepiness, there is great individual variability in the susceptibility to sleepiness in the context of disorders of sleep fragmentation [3] or sleep deprivation [4], which appears to be a stable individual trait. Evidence from several studies indicates that excessive sleepiness is heritable, with heritability estimates from recent twin studies in the range of 0.38–0.48 [5-7]. Persistent circadian rhythm disorders, such as advanced or delayed sleep phase syndrome, are relatively uncommon, estimated to affect <1% of the adult population [8]. However, individual differences in diurnal preference (morning types or "larks" versus evening types or "owls") have important implications for work scheduling and performance that are highly relevant in an economy in which almost one-fifth of employees are engaged in shift work [9]. Both twin and family studies suggest that diurnal preference is heritable, with heritability estimates of 0.23–0.47 for usual bedtime or more formal assessment of diurnal preference [10-12]. Usual sleep duration is an important determinant of daytime sleepiness; moreover, both short and long sleep duration have been associated in numerous epidemiologic studies with hypertension [13], diabetes mellitus [14,15], coronary heart disease [16] and mortality [17-19], although the mechanisms underlying these associations are poorly understood. Significant heritability of usual sleep duration has been reported, with heritability estimates of 0.40–0.44 [10,20].

While sleepiness, diurnal preference, and sleep duration have long been recognized as heritable traits, the genetic basis of this heritability is largely unknown. While it has been suggested that heritability of sleepiness may reflect genetic influences on sleep-disordered breathing [5], sleep drive is itself a highly regulated phenomenon and may be influenced by variations in the numerous genes involved in the circadian and homeostatic regulation of sleep and wakefulness. For example, a polymorphism in the gene encoding adenosine deaminase is reportedly associated with an increase in slow-wave sleep, a marker of homeostatic sleep drive [21]. Similarly, a polymorphism in *HCRTR2*, the gene encoding the orexin/hypocretin receptor 2, has been identified in 2 patients with idiopathic hypersomnolence but in no non-sleepy controls [22]. A polymorphism in the gene encoding this receptor is known to cause autosomal dominant canine narcolepsy [23,24], although the role of polymorphisms in genes of the orexin/hypocretin system in human narcolepsy or daytime sleepiness in general remains uncertain. Polymorphisms in the human period 2 (*PER2*) and casein kinase 1d (*CSNK1D*) genes, known elements of the circadian molecular clock, are associated with autosomal dominant advanced sleep phase syndrome in isolated families [25,26]. A polymorphism in the 3'-untranslated region of the *CLOCK *gene has been inconsistently reported in association with evening preference [27-29] and a length polymorphism in a tandem repeat region of the period 3 protein, containing either 4 or 5 repeats of an 18-amino acid motif, is reportedly associated with diurnal preference [30]. These examples notwithstanding, it appears that sleepiness and diurnal preference are polygenic traits. In a genome-wide linkage analysis of C57BL/6J × BALB/cJ hybrid mice, 14 loci were identified that were significantly linked to circadian phenotypes; only one of these loci was near a gene proposed to be part of the core mammalian circadian clock [31]. We are unaware of any published genome-wide linkage or association studies of daytime sleepiness, diurnal preference, or sleep duration in humans.

The present study takes advantage of sleep phenotype data collected by the Framingham Heart Study at the Offspring Cycle 6 Examination. These data include measures of daytime sleepiness, usual bedtime, usual sleep duration, and sleep-disordered breathing. The aims of this study were to replicate, in this unselected family-based sample, prior reports of heritability of these traits and to conduct genome-wide linkage and association studies of these traits. Although sleep phenotype data were collected from fewer than half of all Offspring participants, heritability of the sleep-related phenotypes was confirmed in this sample and preliminary linkage and association studies identified several loci of interest.

## Methods

Subjects of this study are drawn from the 2848 Framingham Offspring Study participants who completed sleep habits questionnaires between 1995 and 1998 (Offspring Examination Cycle 6) for the Sleep Heart Health Study, a longitudinal study of the cardiovascular consequences of sleep-disordered breathing that has been described elsewhere [32]. Of these subjects, 891 members of 371 pedigrees had biological relatives with valid sleep phenotype data and thus contributed to the heritability analyses. Genome-wide SNP genotyping was performed in the "Family Plate Set" of 1345 members of the largest nuclear families participating in the Framingham Original and Offspring cohorts using an Affymetrix 100K SNP GeneChip, as described in the Framingham Heart Study 100K Project Overview [33]. A maximum of 738 members of 203 families contributed informative data to each of the genetic association analyses of sleep phenotypes.

Data on daytime sleepiness, usual bedtime, and usual sleep duration were obtained from a self-completion questionnaire either handed to the participant at the time of a regularly scheduled visit to the Framingham Study clinic or mailed to the participant. Sleepiness was defined as the score on the Epworth Sleepiness Scale, a widely used and well validated 8-item questionnaire that asks the likelihood of falling asleep in a variety of commonly encountered situations [34,35]. Usual bedtime was obtained from the single question, "What time to you usually go to bed on weeknights (or work nights)?" Usual sleep duration was obtained from the single question, "How many hours of sleep do you usually get on weeknights (or work nights)?" with integer response options. The full Sleep Habits Questionnaire is available from the Sleep Heart Health Study website [36]. Data on work shift and retirement status were not available in this cohort; therefore, in order to exclude subjects in whom night shift work might lead to spurious estimates of circadian phenotype, the 0.5% of subjects reporting a usual bedtime between 5 AM and 6 PM were excluded from analyses of usual bedtime and usual sleep duration. Those whose usual bedtime differed by more than two hours between weekdays and weekends (1.3%) were excluded from analyses of usual bedtime, as behavioral factors were presumed to have a major influence on this measure. Similarly, those whose usual sleep duration differed by more than two hours between weekdays and weekends (4.2%) were excluded from analyses of sleepiness and usual sleep duration. As sleepiness, bedtime, and sleep duration may be influenced by age, sex and BMI [5,10,20], adjustment for these variables was made by linear regression. Standardized residuals of the adjusted sleep phenotype variables were used in genetic analyses. As further adjustment of sleepiness for usual sleep duration or self-reported symptoms of sleep-disordered breathing (snoring, nocturnal breathing pauses) reduced sample size but had little impact on the linkage and association results, analyses using these further adjustments are not presented. Standardized residuals were obtained using PROC REG in the SAS statistical software package (SAS version 9.1, SAS Institute, Cary, NC).

Heritability, linkage and genetic association analysis was performed as described in the Framingham Heart Study 100K Project Overview [33]. Briefly, multipoint linkage analysis was implemented using Merlin identity-by-descent estimates and variance component linkage in SOLAR with a subset of 10,588 SNPs supplementing 612 microsatellite markers from a previous genome scan. Association of SNPs to sleep phenotypes was studied with both population-based association tests using generalized estimating equations (GEE) and family-based association tests (FBAT). All association tests employed an additive model. Reported association test results are limited to SNPs located on autosomes and meeting the following quality control criteria: minor allele frequency of ≥10%; call rate ≥80%; and no significant deviation from Hardy-Weinberg equilibrium (p ≥ 0.001). Of the 70,987 SNPs meeting these criteria, 40,249 were located within 60 kb of a known or putative gene. Physical locations are based on National Center for Biotechnology Information build 35.

Of the 2848 Offspring Study participants who completed the sleep habits questionnaire, 699 also underwent overnight polysomnography; therefore, polysomnographic data on sleep-disordered breathing were available in a small subset of subjects included in the Family Plate Set (n = 219). There were too few subjects to permit linkage analysis and, as power was very low for association studies, these data are not considered in this manuscript but are posted on the web site. The correlation of sleepiness, bedtime, and sleep duration with polysomnographically measured apnea-hypopnea index was weak (correlation coefficients -0.07 to 0.10).

## Results

Sleep characteristics of the 749 subjects included in the Framingham Study Family Plate Set with available sleep phenotype data are shown in Table 1. Mean age was 55.8 (SD 9.4) years, mean BMI was 28.2 (SD 5.5) kg/m^2^, and 51.5% were women. Heritability of sleepiness was 0.29 (p < 0.001), of usual bedtime 0.22 (p < 0.01), and of usual sleep duration 0.17 (p = .02). The estimated heritability of sleepiness was not appreciably reduced by further adjustment for usual sleep duration and the presence of habitual snoring or witnessed apneas (0.28, p = 0.002). Linkage analysis to these three phenotypes identified no linkage peaks with LOD >3. Five linkage peaks with LOD >2 were identified, four to usual bedtime and one to usual sleep duration (Table 2c). The region of the strongest of these, a LOD score of 2.45 for usual bedtime with a linkage peak at 55.5 Mb on chromosome 16, includes the gene encoding casein kinase 2a2 (*CSNK2A2*, at 56.7 Mb), a known element of the Drosophila circadian clock. The linkage peak to usual sleep duration at 71.3 Mb on chromosome 3 includes the gene encoding prokineticin 2 (*PROK2*, at 71.9 Mb), which may be an important output molecule from the suprachiasmatic nucleus. Three additional linkage peaks to usual bedtime had maximum LOD scores of 1.9. One of these, located on chromosome 2, includes the interleukin-1 cytokine cluster. Another, located on chromosome 4 at 62.5 Mb, includes the *CLOCK *gene (at 56.0–56.1 Mb). A smaller linkage peak of LOD 1.5 was noted in this region (at 57.1 Mb) for sleepiness. Kurtosis was low for sleepiness and usual bedtime, at 0.2 and 1.1, respectively, although kurtosis of usual sleep duration was high at 3.0.

**Table 1:** Sleep phenotypic characteristics of Family Plate Set subjects

Phenotype	N	Offspring Exam cycle	Unadjusted Mean (SD)	Adjustment variables	Heritability (SE)	Webpost name*
Epworth Sleepiness Scale score	721	6	6.4 (3.8)	age, sex, BMI	0.29 (0.09)	*essresid1*
Usual bedtime, (hr:min)	738	6	10:50 PM (1:06)	age, sex, BMI	0.22 (0.08)	*bedtimeresid*
Usual sleep duration, hrs	736	6	7.28 (1.06)	age, sex, BMI	0.17 (0.08)	*sleepdurresid*

*The age-, sex- and BMI-adjusted residual apnea-hypopnea index (*rdiresid*) is also posted in the on-line repository, although it is not discussed in this manuscript. Also posted in the on-line repository but not included in this manuscript are the unadjusted Epworth Sleepiness Scale score (*ess*), usual bedtime (*bedtime*), and usual sleep duration (*sleepdur*), as well as Epworth Sleepiness Scale score additionally adjusted for usual sleep duration (*essresid2*) and for both usual sleep duration and the presence of self-reported sleep-disordered breathing symptoms of snoring three or more nights per week or any witnessed nocturnal apneas (*essresid3*).

**Table 2:** Top genetic association and linkage results

2a – Top association results from population-based association tests						
**Phenotype**	**SNP**	**Chr**	**Physical Position**	**GEE p-value**	**FBAT p-value**	**Gene Region**

Sleepiness	rs1823068	5	58,711,806	2.5*10^-8^	.069	*PDE4D*
Sleepiness	rs2218488	8	72,426,510	3.2*10^-6^	.070	*EYA1*
Sleepiness	rs2247614	21	35,553,557	.000011	.0045	
Sleepiness	rs7073579	10	1,364,007	.000014	.0081	*ADARB2*
Sleepiness	rs9298693	9	13,891,409	.000016	.28	
Bedtime	rs949175	11	97,242,217	9.7*10^-6^	.38	
Bedtime	rs2288292	11	12,452,275	.000017	.0096	*PARVA*
Bedtime	rs324981	7	34,591,353	.000018	.089	*NPSR1*
Bedtime	rs10483871	14	75,141,034	.000045	.0065	*C14orf58*
Bedtime	rs10507551	13	46,659,884	.000048	.032	
Sleep duration	rs6599077*	3	40,071,622	1.4*10^-7^	.020	*MYRIP*
Sleep duration	rs10492604	13	57,802,314	4.2*10^-6^	.038	
Sleep duration	rs10489832	1	155,267,429	.000028	.0043	*OR10K1, OR10K2*
Sleep duration	rs2256551	20	51,066,908	.000065	.11	*TSHZ2*
Sleep duration	rs2165207	12	72,418,535	.000011	.13	

**2b – Top association results from family-based association tests**						

**Phenotype**	**SNP**	**Chr**	**Physical Position**	**GEE p-value**	**FBAT p-value**	**Gene Region**

Sleepiness	rs10510835	3	60,222,161	.00054	.000019	*FHIT*
Sleepiness	rs2189829	7	49,387,124	.53	.000019	
Sleepiness	rs434052*	14	35,613,399	.0024	.000024	
Sleepiness	rs4896580	6	142,518,529	.000096	.000056	*VTA1*
Sleepiness	rs10520010	4	150,482,987	.013	.000064	
Bedtime	rs1940013*	11	131,786,861	.0026	6.1*10^-6^	*OPCML*
Bedtime	rs2525724	7	120,203,894	.33	.000019	*ING3*
Bedtime	rs1725021	7	136,350,575	.031	.000022	*PTN*
Bedtime	rs932650	10	115,337,349	.37	.000025	*HABP2*
Bedtime	rs2985334	1	29,165,643	.0099	.000035	*EPB41*
Sleep duration	rs481233	6	126207158	.85	.000072	*NCOA7*
Sleep duration	rs2359894	18	40309270	.059	.00011	
Sleep duration	rs2061579	12	73224522	.13	.00014	
Sleep duration	rs10492507	13	48722291	.037	.00021	*CDADC1*
Sleep duration	rs6974138	7	74708138	.20	.00024	

**2c – Linkage peaks with LOD score >2.0**						

**Phenotype**	**SNP closest to linkage peak**	**Chr**	**Physical location of linkage peak**	**Maximum LOD score**	**1.5 LOD support interval (Mb)**	

Bedtime	rs28168	16	55,508,777	2.45	48.5 – 62.0	
Bedtime	rs10498313	14	29,468,627	2.33	28.7 – 32.6	
Bedtime	rs10503857	8	29,909,250	2.26	21.9 – 37.1	
Bedtime	rs10512058	9	76,736,108	2.11	10.6 – 107.4	

*A second strongly associated SNP in this gene region is not reported in the table because it is in close proximity to and in linkage disequilibrium (D' 1.0) with the reported SNP.

The five autosomal SNPs with the lowest p-value for association for each sleep phenotype by population-based and family-based association tests, and meeting quality control standards, are displayed in Tables 2a and 2b. Only one of these is located in a coding region: rs324981 in *NPSR1*, associated with usual bedtime, whose minor allele (frequency 0.44) is a non-synonymous mutation encoding an Asn^107^→Ile^107 ^substitution in an exoloop lining the putative ligand-binding pocket of the neuropeptide S receptor [37]. The effect of this polymorphism is additive, with adjusted mean bedtime delayed by 14.9 minutes in heterozygotes and 29.5 minutes in homozygotes. Only eight SNPs were associated with any of the sleep phenotypes with p < 10^-5^. The SNP with the lowest p value for association to any of the sleep phenotypes is for the association of rs1823068 with sleepiness by population-based association testing (p = 2.5*10^-8^). This SNP is located in an intron of the gene encoding phosphodiesterase 4D (*PDE4D*). Other SNPs associated at p < 10^-5 ^include one located in an intron of the gene encoding eyes absent 1 (*EYA1*) and two within an intron of the gene encoding myosin VIIA and Rab interacting protein (*MYRIP*) identified by population-based association tests, and two in or near the gene encoding opioid binding protein/cell adhesion molecule (*OPCML*) by family-based association tests. One was not located near a known gene.

The results of population-based and family-based association tests were modestly correlated. For example, when SNPs were ranked based on the p value for association with sleepiness, the Spearman correlation coefficient for rankings from population-based versus family-based tests was 0.22. Tables 2a and 2b show p-values for both approaches, allowing assessment of concordance across approaches for these SNPs. Only 3 SNPs were associated with sleepiness at p < 0.001 by both approaches; these were located in *FHIT, VTA1*, and *LRP1B*. Of the 5 SNPs meeting this criterion for usual bedtime, none was in or near a known gene, and no SNPs met this criterion for usual sleep duration. Similarly, little overlap was seen across the three phenotypes. No SNPs associated with sleepiness at p < 0.001 were associated with either usual bedtime or sleep duration at this nominal significance level for either population-based or family-based tests, and only 4 SNPs met this criterion for overlap between usual bedtime and sleep duration; three were located in known genes *RYR2*, *JAZF1*, and *NDRG1*.

Among potential candidate genes for sleep-related phenotypes, many were poorly represented on the Affymetrix 100K GeneChip. No SNP meeting quality control standards was located within *PROK2*, identified in the linkage analysis as a possible candidate for usual sleep duration. Only a single SNP was located in *PER2 *and none in *CSNK1D *or *PER3*, genes associated with familial advanced or delayed sleep phase syndromes. In contrast, 10 SNPs meeting quality control standards were typed within the *CLOCK *gene and 5 within *CSNK2A2*, none of which was significantly associated with any sleep phenotype by either the population-based or family-based approaches (lowest p value 0.12). The full results of the genetic association studies are available at the National Center for Biotechnology Information dbGaP website [38].

## Discussion

In this family-based study, we have confirmed significant heritability of sleepiness, usual bedtime, and usual sleep duration that had been previously reported primarily from twin cohorts [5-7,10-12,20]. The heritability estimates in this study are lower than those reported in the literature from twin studies. This may reflect a greater contribution of environmental influences on these phenotypes in the present study that is detected as correlation between spouse-pairs. Alternatively, an underestimate of the shared environmental variance in twin studies may cause them to overstate the genetic contribution, as the estimated heritability of usual bedtime in this study is similar to that for diurnal preference in a previous family-based study [12]. We are unaware of any prior genome-wide linkage or association studies of these sleep-related phenotypes in humans. Because sleep phenotypes were available from only 56% of subjects included in the Framingham Family Plate Set, study power was limited and no linkage peaks reaching conventional levels of genome-wide significance were observed. Despite the greater heritability of sleepiness, most of the suggestive linkage peaks observed in this study were linked to usual bedtime and to a lesser extent to usual sleep duration. Several of these suggestive linkage peaks contain genes of potential importance to the circadian molecular clock. Two of these peaks are of particular interest. The linkage to usual bedtime on chromosome 16 was the strongest observed in this study (LOD = 2.45), with a peak close to the gene *CSNK2A2*. Its product, a catalytic subunit of casein kinase 2, has been shown to be an important component of the circadian molecular clock in Drosophila and other organisms [39]. Phosphorylation by casein kinase 2 promotes nuclear translocation of the *PERIOD *gene product, and mutations that impair catalytic activity or subunit multimerization cause a lengthening of the circadian period [40,41]. Although casein kinase 2 has not previously been implicated in human circadian rhythm disorders, mutations in the human genes encoding casein kinase 1d and period 2 are associated with familial advanced sleep phase syndrome [25,26]. The linkage to usual sleep duration on chromosome 3 (LOD = 2.17) has a peak close to the gene *PROK2*. Its product is the precursor of prokineticin 2, which is highly expressed in the suprachiasmatic nucleus, regulated by the circadian molecular clock, and believed to be an important output molecule from the suprachiasmatic nucleus, coordinating and transmitting the behavioral circadian rhythm to multiple brain regions [42,43]. Although not previously implicated in human disorders, the total sleep duration of prokineticin null mice is reduced by 83.5 minutes per 24 hour period compared to their wild-type littermates [44]. The modest linkage to both bedtime and sleepiness near the *CLOCK *gene, a central component of the molecular circadian clock, is also intriguing. *PROK2 *could not be evaluated in association tests, as no SNPs were typed within this gene. As none of the 5 SNPs within *CSNK2A2 *or the 10 within *CLOCK *was significantly associated with usual bedtime, other genes in these regions or chance may be responsible for these linkage peaks.

Association tests identified several loci that merit follow-up in other cohorts. The most interesting of these is an association of usual bedtime with a non-synonymous coding SNP in *NPSR1*, which causes an Asn^107^→Ile^107 ^substitution in the putative ligand-binding pocket of the neuropeptide S receptor [37]. This same variant has been linked to asthma in several Caucasian populations [45,46] but has not been previously reported in association with any sleep or circadian phenotype. In mice, neuropeptide S is localized to a small area adjacent to the noradrenergic locus ceruleus and its intraventricular administration is a potent, transient stimulus to wakefulness [47]. The Asn^107^→Ile^107 ^variant of the neuropeptide S receptor is a gain of function mutation, increasing sensitivity of the receptor to neuropeptide S [37]. Consistent with this effect on receptor function, mean bedtime is 15 minutes later for each copy of the gene encoding the Asn^107^→Ile^107 ^variant.

Although not located in a coding region, the strong association of sleepiness with a SNP located in an intron of the gene encoding phosphodiesterase 4D also merits further study. Phosphodiesterase 4 is a cAMP-specific phosphodiesterase that has multiple splice variants, with PDE4D being widely expressed in human brain [48]. Mutations of *PDE4D *have been associated with stroke risk in several populations, possibly related to the role of PDE4 in modulating inflammatory processes, although the causal nature of the association remains controversial [49,50]. While the nonselective phosphodiesterase inhibitors caffeine and theophylline have long been recognized to promote wakefulness, this is likely due to antagonism of dopamine receptors rather than phosphodiesterase inhibition [51]. However, variation in the effects of PDE4D on brain intracellular levels of cAMP or extracellular levels of adenosine might influence sleepiness, and the selective PDE4 inhibitor rolipram is a weak promoter of wakefulness in rats [52].

This study has a number of limitations. The sleep phenotypes were assessed by questionnaire only. While the Epworth Sleepiness Scale is a well-validated measure of usual sleepiness, the single questions regarding usual bedtime and sleep duration provide only crude measures of circadian phenotype. Moreover, as subjects did not have a clinical sleep evaluation, it was not possible to control for sleep apnea or other primary sleep disorders. A sleepiness phenotype further adjusted for self-reported usual sleep duration and frequent snoring or witnessed apneas was analyzed, however, and results are included in the web repository. This gave results very similar to those presented in this manuscript, although with somewhat lower power due to an additional 15% missing phenotype data. The present study has statistical limitations as well. The relatively small number of subjects included in the analysis limits the power to detect true linkage and association, while the large number of SNPs tested and apparent inflation of Type I error rates for the population-based association tests makes it likely that many of the observed associations are false positive findings. Kurtosis of the measure of sleep duration may have inflated the LOD scores from linkage analysis. Thus, all results of this study require replication in other populations. These statistical limitations are discussed in more detail in the Overview [33].

Notwithstanding these limitations, this study begins to apply the powerful methodologies of genetic epidemiology to the study of common sleep and circadian phenotypes, and identifies for further study several genes that have not previously been implicated in human sleep and circadian disorders. These findings require replication, which will be pursued in other cohorts and by a planned expansion of the SNP genotyping using a 500,000 SNP gene chip in a substantially larger sample of Framingham Study subjects. Collection of more detailed sleep phenotype data from a larger sample of Framingham Study subjects will increase the power to detect novel genes influencing sleep and circadian phenotypes.

## Conclusion

This analysis confirms, in a family-based sample, prior reports of significant heritability of sleepiness, usual bedtime, and usual sleep duration. It identifies several genetic loci with suggestive linkage to these traits, including linkage peaks containing the circadian clock-related genes *CSNK2A2*, *PROK2 *and *CLOCK*. Among genes identified by association tests as possible mediators of sleep and circadian phenotypes, those most promising based on the strength of the associations and their known biological activity are *NPSR1 *and *PDE4D*, which may influence usual bedtime and daytime sleepiness, respectively. While these findings require replication in other samples, they provide evidence of the possible utility of genetic epidemiology approaches to understanding population variation in sleep and circadian phenotypes.

## Competing interests

The authors declare that they have no competing interests.

## Authors' contributions

All authors participated in the conception and design of the study. DJG and GTO participated in phenotype data collection. DJG and JBW participated in the statistical analysis. DJG drafted the manuscript, with critical feedback from GTO and JBW. All authors read and approved the final manuscript.
